# A Tale of Two Infections: A Rare Synchronous Infection of Herpes Simplex Virus Oesophagitis and Clostridium difficile in an Immunocompetent Host

**DOI:** 10.7759/cureus.94660

**Published:** 2025-10-15

**Authors:** Sujata Lama, Aye Thinzar Moe, Amirat Akinloye, Kyaw Soe Tun, Jithin Karedath

**Affiliations:** 1 General Medicine, King's College Hospital NHS Foundation Trust, London, GBR; 2 Stroke Medicine, Dartford and Gravesham NHS Trust, Dartford, GBR; 3 Geriatrics, King's College Hospitals NHS Foundation Trust, London, GBR; 4 Pathology, Southmead Hospital, Bristol, GBR

**Keywords:** elderly population, herpes esophagitis, immunocompetent adult, immunosenescence, proton-pump inhibitors (ppi), recurrent clostridium difficile infection

## Abstract

Herpes simplex virus (HSV) is a common cause of infectious oesophagitis, predominantly affecting immunocompromised populations. *Clostridium difficile *(*C. difficile*)*, *while prevalent in this population, is also a frequently encountered cause of nosocomial diarrhoea associated with antibiotic exposure, prolonged hospitalisation, or residence in long-term care facilities. A simultaneous HSV oesophagitis and *C. difficile *infection (CDI), despite the absence of the typical risk factors, is rare but possible, as illustrated by the following case.

A 95-year-old female patient presented with acute confusion, abdominal pain, diarrhoea, odynophagia, and dysphagia. The stool sample detected toxigenic *C. difficile*, and a course of fidaxomicin was completed. Oesophagogastroduodenoscopy (OGD) observed mucosal changes consistent with HSV oesophagitis, but treatment was initially withheld due to pending histopathology. She was readmitted due to the recurrence of her symptoms. HSV was confirmed, and the patient started on dual antimicrobial therapy with marked improvement and resolution.

This case study prompts us to consider non-traditional risk factors such as immunosenescence, frailty, proton pump inhibitors (PPIs), and nutritional deficiencies in addition to the lack of primary prevention measures.

## Introduction

Herpes simplex virus (HSV) is an opportunistic pathogen and a common cause of infectious oesophagitis. HSV infects the visceral organ via latent reactivation with spread along the vagus nerve or when an oropharyngeal primary infection directly extends to the oesophageal mucosa ​[1, 2]. *Clostridium difficile* (*C. difficile*) is a nosocomial gram-positive anaerobic bacterium linked to gastrointestinal dysbiosis and can manifest from asymptomatic carriage to gastrointestinal symptoms ​[3]​. 

HSV oesophagitis is reported almost entirely in immunocompromised populations such as those with haemato-oncological neoplasms, organ transplantation, and prolonged use of corticosteroids ​[4]​. While *C. difficile* infections (CDI) are frequent and with increased recurrence rates in these groups, they also encompass additional predisposing factors, particularly exposure to antibiotics and the organism, typically via hospital or residential home admissions ​[5]. A concurrent infection with these pathologies in an immunocompetent host is rarely observed, with no prior cases reported in the literature to our knowledge. Furthermore, due to the overlapping gastrointestinal symptoms, it may lead to a delay in management, potentially leading to poorer clinical consequences. Therefore, we present a case of a 95-year-old female patient with recurrent admissions related to concomitant HSV oesophagitis and CDI, inviting us to explore atypical contributors and diagnostic dilemmas.

## Case presentation

First admission 

A 95-year-old female patient presented with a three-week history of abdominal pain, watery diarrhoea, and new-onset confusion. Additionally, she reported progressive odynophagia and dysphagia with reduced oral intake in the last one to two months. She was previously independent, resided alone, and mobilised interchangeably with a walking stick or Zimmer frame. She denied any alcohol intake, smoking, or substance abuse. Her past medical history included gastro-oesophageal reflux disease (diagnosed two years prior, managed with regular omeprazole), hypothyroidism (on replacement therapy), osteoarthritis, and diverticular disease. There was no recent exposure to immunosuppressive medications, corticosteroids, antibiotics, or hospitalisation. 

On examination, she was dehydrated and hypothermic (35.6°C) and had mild suprapubic and left upper quadrant abdominal tenderness. The rest of the examination was unremarkable. Laboratory parameters indicated leucocytosis with neutrophilic predominance, elevated CRP, acute kidney injury (AKI) stage 2, and hypoalbuminemia (Table 1). Venous blood gas revealed hyperchloremic non-anion gap metabolic acidosis, likely in the context of gastrointestinal depletion. There was clinical improvement with intravenous fluid resuscitation and other supportive therapy. 

**Table 1:** Laboratory investigations in the first admission

Parameter	Result	Reference ranges	Interpretation
White blood cells (WBC)	11.8 × 10⁹/L	2.9 - 9.6 × 10⁹/L	↑ Leucocytosis with neutrophilic predominance
Neutrophils	9.29 × 10⁹/L	1.5 - 6.10 × 10⁹/L	
C-reactive protein (CRP)	315 mg/L	<5 mg/L	↑ Markedly elevated
Estimated glomerular filtration rate (eGFR)	21 mL/min/1.73 m2	> 90 mL/min/1.73 m2	↑ Acute kidney injury stage 2
Creatinine	177 µmol/L	49 – 90 µmol/L	
Albumin	28 g/L	31 – 45 g/L	↓ Hypoalbuminemia
Alkaline phosphatase (ALP)	184 U/L	30 – 130 U/L	↑ Elevated
Gamma-glutamyl transferase (GGT)	58 U/L	<38 U/L	↑ Elevated
Total bilirubin	15 µmol/L	<21 µmol/L	Normal

Stool samples detected the presence of glutamate dehydrogenase (GDH), and confirmatory polymerase chain reaction (PCR) identified toxigenic C. difficile. Notably, exotoxins A and B were not detected, but due to the symptomatic condition, a 10-day oral fidaxomicin course was commenced, and omeprazole was switched to famotidine in accordance with local guidelines. Other acute diarrheagenic infectious aetiologies, such as astrovirus, norovirus G1 and G2, rotavirus, sapovirus, adenovirus, faecal cultures and microscopy for *Cryptosporidium*, were negative. 

**Figure 1:**
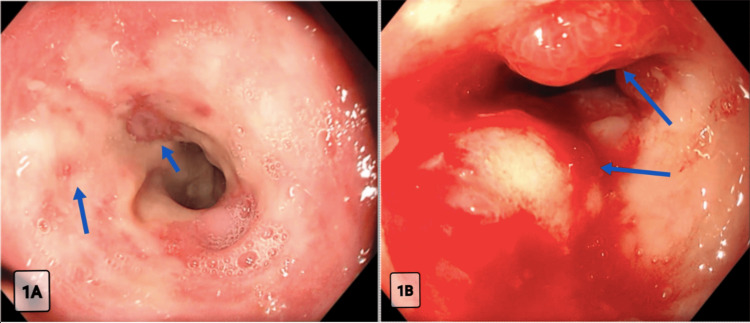
(A) OGD showing multiple discrete punched-out ‘volcanic’ ulcers, oedematous mucosa, plaque, and loss of vascular pattern in the distal oesophagus; (B) Friable, oedematous mucosa with fresh bleeding and early stricturing. OGD: oesophagogastroduodenoscopy

During the admission, a formal swallow assessment identified odynophagia localised to the thoracic midline with intact oropharyngeal function. An oesophagogastroduodenoscopy (OGD) visualised multiple ‘punched out' ulcers and oedematous mucosa with extensive plaque in the lower third of the oesophagus (Figure 1A). Additionally, a circumferential ulceration at the distal oesophagus with minimal stricturing and fresh bleeding was observed (Figure 1B). Cold forceps biopsies were retrieved for further investigations, including the exclusion of malignancy. With improved oral intake and resolution of her diarrhoea, AKI, and confusion, she was discharged after eight days of hospitalisation with fidaxomicin, and an outpatient follow-up was arranged.

Interim period 

**Figure 2:**
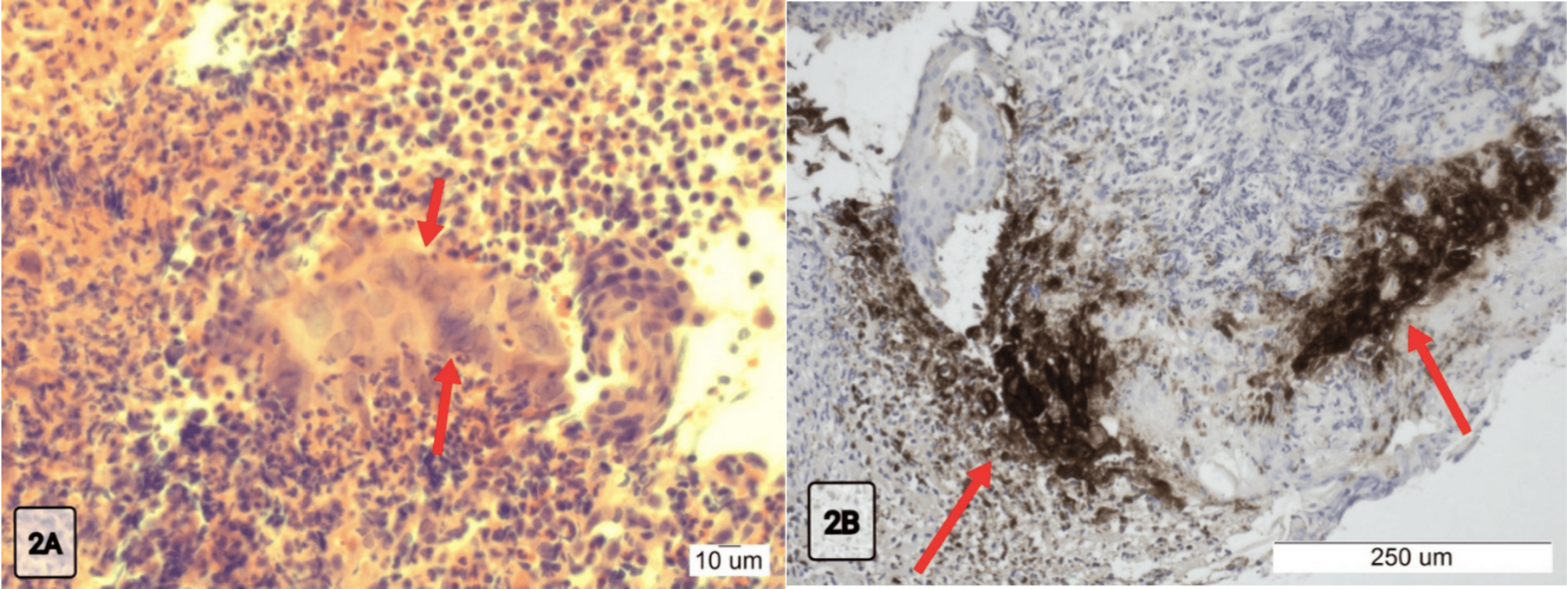
(A) H&E staining, multinucleated squamous cells with nuclear moulding, inclusion bodies, chromatin margination and ground glass appearance; (B) Immunohistochemical staining, positive staining of HSV-infected squamous cells HSV: herpes simplex virus

Histological examination of the ulcer margin identified the characteristic viral cytopathic changes consistent with HSV infection (Figure 2A). This was confirmed by immunohistochemical staining (Figure 2B), and no neoplastic changes were identified. Biopsy results were acknowledged in a virtual outpatient follow-up; however, the patient was readmitted before antiviral therapy could be implemented. 

Second admission 

One month later, the patient re-presented to the emergency department due to functional decline secondary to recurrence of watery diarrhoea and new-onset faecal incontinence, which worsened to occur during any positional changes. Her home environment was notably cluttered with extensive soiling of clothing, furniture and floors. The subsequent CDI associated with anorexia, malaise and dehydration contributed to the reduced mobility and profound deconditioning. She had been compliant with the previous fidaxomicin regimen, which had initially subsided her symptoms. Physical examination, including abdominal assessment, was unremarkable. Laboratory parameters showed leucocytosis, elevated CRP, hypoalbuminemia, mild hypokalaemia, and hypophosphatemia (Table 2). 

**Table 2:** Laboratory Investigations in the second admission

Parameter	Result	Reference ranges	Interpretation
White blood cells (WBC)	14.4 × 10⁹/L	2.9 - 9.6 × 10⁹/L	↑ Leucocytosis with neutrophilic predominance
Neutrophils	11 × 10⁹/L	1.5 - 6.10 × 10⁹/L	
C-reactive protein (CRP)	173 mg/L	<5 mg/L	↑ Elevated
Estimated glomerular filtration rate (eGFR)	68 mL/min/1.73 m2	>90 mL/min/1.73 m2	Normal
Creatinine	66 µmol/L	49 – 90 µmol/L	
Albumin	25 g/L	31 – 45 g/L	↓ Hypoalbuminemia
Alkaline phosphatase (ALP)	92	30 – 130 U/L	Normal
Gamma-glutamyl transferase (GGT)	22	<38 U/L	
Total bilirubin	15	<21 µmol/L	
Potassium	3.3 mmol/L	3.5 - 5.3 mmol/L	↓ Mild hypokalaemia
Phosphate	0.74 mmol/L	0.80 - 1.50 mmol/L	↓ Mild hypophosphatemia

The microbiology team recommended a tapering regimen of vancomycin in accordance with the Infectious Disease Society of America (IDSA) guidelines, with repeat stool cultures. These cultures were subsequently positive for GDH and toxigenic *C. difficile*, as well as exotoxins A and B. CT imaging of the abdomen and pelvis did not identify any CDI complications or alternative pathology accounting for her symptoms. Acute serology for HSV-1 and HSV-2 and cytomegalovirus PCR was unremarkable; however, she developed a recurrence of odynophagia and delirium, prompting consultation with the virology team. After a multidisciplinary discussion, a seven-day course of oral valacyclovir was initiated. Simultaneous physiotherapy aided her to regain strength, and eventually she was able to mobilise with a walking aid independently, returning to her baseline functional status. Following resolution of her symptoms, she was discharged with continuation of antimicrobial therapy. 

Subsequent course 

The patient was discharged back to the care of her general practitioner in the community. Incidentally, she was admitted one month later due to a mechanical fall, unrelated to her previous condition. There was adequate resolution of symptoms, normal biochemical markers, and her functional status was maintained. 

## Discussion

The presence of dual HSV oesophagitis and CDI in a presumed immunocompetent host is a rare finding. In addition, the simultaneous infection, in the absence of non-traditional risk factors such as recent antibiotic exposure (the last confirmed course being flucloxacillin 1.5 years earlier for lower limb cellulitis), HIV/AIDS, or post-transplant setting, poses important questions regarding alternative host susceptibilities and pathophysiological processes ​[6]​. Its recognition is clinically significant, as overlapping symptoms may obscure the diagnosis, resulting in delayed management, preventable complications, prolonged and recurrent hospitalisations, and the prevention of the execution of prophylactic measures. To our knowledge, published literature describing these processes is extremely limited, and this case aims to contribute to an underdeveloped area by bringing light to the potential role of several predisposing factors, including immunosenescence, frailty, proton pump inhibitors (PPIs), and nutritional deficiencies that may have contributed to her susceptibility. 

Immunosenescence is the diminishing of immune function with advancing age, resulting in higher rates of infection, malignancies, and autoimmune diseases ​[7]​. This process involves a decline in T-cell output, chronic low-grade inflammation and thymic involution. When combined with frailty (Frailty Index 0.25) associated with the diminishing of physiological reserve, it likely magnified our elderly patient’s vulnerability ​[8,9]​. Additionally, the patient had chronic PPI exposure (omeprazole) for gastro-oesophageal reflux symptoms, which in hindsight may have represented initial symptoms of HSV oesophagitis. PPIs are also strongly associated with a two-fold increase in CDI due to alkalisation of the colonic environment and disruption of the gut microbiome. It is hypothesised that PPIs contribute to dysbiosis by amplifying the proliferation of *C. difficile* and by disturbing the ability of the normal microbiome to suppress pathogenic organisms ​[10]. Once *C. difficile* was identified, omeprazole was switched to famotidine, as histamine type 2 receptor antagonists (H2RAs) have a significantly lower association with CDI ​[11]​. 

Furthermore, nutritional deficiencies in the form of weight loss, hypoalbuminemia, and electrolyte derangement were a prominent feature in this case. These were precipitated by HSV oesophagitis-related manifestations and amplified by the CDI. Poor nutritional status is consistently correlated with reduced immune competence and homeostasis and likely contributes to disease progression ​[12]​. Given the role of albumin as a negative acute phase protein and in protein loss enteropathy, hypoalbuminemia is recognised as a predictor for severity and recurrence of CDI ​[13]​. The patient met the criteria for severe CDI, signifying a higher risk for complications and relapses. An early recognition of this relationship could have guided risk stratification, diagnostics, and management ​[14]​. 

Both pathologies are comparable in terms of diagnostic approach. HSV oesophagitis has distinctive endoscopic features ranging from erosive changes to well-circumscribed ‘volcanic’ ulcers, occasionally with plaques and exudates ​[15,16]​. In this case, the OGD features were consistent with these changes, which affected the distal oesophagus. Histological sections from the ulcer edge revealed squamous epithelial cells characterised by multinucleation, nuclear moulding, and prominent ground-glass intranuclear viral inclusion bodies with chromatin margination. The HSV infection was confirmed with immunohistochemical staining. Importantly, HSV-1 and HSV-2 serologies were negative during these admissions, likely reflecting a localised infection but reinforcing histopathological diagnosis as the gold standard for HSV oesophagitis ​[15]​. 

Similarly, investigations for CDI should only be employed in symptomatic patients, given the high prevalence of asymptomatic carriage, and current recommendations support a multistep algorithm ​[17]​. Systematic reviews demonstrate exotoxins A and B assays have a lower sensitivity and specificity than PCR ​[5]​. In this case, both the GDH enzyme and PCR for toxigenic *C. difficile* were positive, but the absence of exotoxins A and B implied colonisation if considered in isolation. However, due to active clinical manifestations, antibiotics were indicated. This reflects current practice, which discourages sole reliance on exotoxins A and B assays for diagnosis and emphasises the importance of integrating clinical context when interpreting isolated data for CDI. 

Clinical context was equally crucial in the management of HSV oesophagitis. Despite the diagnostic confirmation, antivirals were initially withheld due to the asymptomatic state, as HSV is typically self-limiting in immunocompetent patients ​[1]​. When the patient later developed odynophagia and dysphagia, treatment with valaciclovir proved to be beneficial. Valaciclovir was particularly advantageous over aciclovir, as it offers equal efficacy with a less frequent dosing schedule in our frail patient with swallowing difficulties. 

Management of severe CDI infection proved to be more challenging due to its recurrence and significant impact on quality of life. Fidaxomicin, vancomycin, and metronidazole are the most common antibiotic therapies for CDI, each with varying recurrence rates. Current literature demonstrates that fidaxomicin is associated with significantly lower relapse rates than vancomycin and metronidazole in both severe and non-severe disease; therefore, it is recommended as first-line therapy by the IDSA guidelines ​[17]​. In this case, due to the relapse despite fidaxomicin, a tapering regimen of vancomycin was commenced in line with IDSA recommendations ​[18]. 

CDIs are linked to considerable morbidity, mortality, and healthcare costs, with a 13.5% mortality rate in patients over 80 years of age ​[19]. Antibiotic stewardship, together with rapid and more accurate diagnostics, has contributed to an overall decline in the healthcare burden. While virtually all antibiotics are associated with CDI, clindamycin, later-generation cephalosporins, and fluoroquinolones pose the greatest risk. Restrictive prescribing practices have been strongly correlated to reduced *C. difficile* incidence and colonisation ​[14]​. Furthermore, rapid diagnostic tests such as nucleic acid amplification tests have enabled early detection, prompt treatment, and implementation of secondary prevention strategies, including isolation and environmental decontamination. However, this has led to an increase in the identification of asymptomatic carriage. While previously considered protective against symptomatic CDI, emerging evidence now suggests a higher risk of progression to symptomatic disease ​[20]​. This signals the potential role colonisation status could have as a predictive marker, particularly in geriatric patients. 

Conversely, HSV infections remain significantly more challenging with no real population-level prevention measures beyond prophylactic antivirals offered to immunocompromised patients. This is mainly attributed to the virus’s latent characteristics ​[2]​. Primary prevention methods for both pathologies remain limited, with immunoprophylaxis not advancing beyond clinical trials, though epidemiological models for *C. difficile* vaccines have indicated benefits when targeting elderly populations [14, 18].

## Conclusions

This case illustrates the complex interplay in an ageing immune system between physiology, pharmacodynamics, and nutritional status, which collectively render the host more susceptible to infections. Past the diagnostic novelty, the occurrence of HSV oesophagitis and CDI in a presumed immunocompetent patient identified the need for exploration into non-conventional risk factors. It also reinforced the multidisciplinary and individualised approach required for diagnostics, management, and prevention strategies. 

A patient-centred viewpoint remained a cornerstone in this case, mirroring the principles in geriatrics, with care extending beyond biochemical, endoscopic, and histopathological features. A targeted approach, such as valaciclovir, chosen for its reduced dosing schedule, and symptom-based initiation of antimicrobials, was a central theme for effective treatment of the concurrent infection. This is particularly important in the elderly population, where the functional decline, rehospitalisations, and multiple antimicrobial therapies carry their own risks and complications. Additionally, the scarcity of proven evidence-based measures for recurrent and dual infections highlights the current gap in biogerontology. As this report is limited to a single patient, it calls for further research with a more significant sample size to develop effective primary prophylaxis, treatment strategies, and screening mechanisms, seeking to improve clinical outcomes and guide patient-centred care, especially in this vulnerable population. 
